# Non-thermal venous endothelial injury by microsecond irreversible electroporation: mechanisms of apoptosis, oxidative stress, and vascular closure initiation

**DOI:** 10.3389/fcell.2026.1887504

**Published:** 2026-07-15

**Authors:** Jiang Xu, Xiaotian Wang, Fubao Liu, Qiang Chen, Yao Zhang, Can Cheng

**Affiliations:** 1 The First Affiliated Hospital of Anhui Medical University, Hefei, China; 2 The First Affiliated Hospital of University of Science and Technology of China, Hefei, China; 3 Zhejiang Curaway Enterprise Research Institute of Intelligent Minimally Invasive Precision Therapy, Hangzhou, China

**Keywords:** apoptosis, damage-associated molecular patterns, irreversible electroporation, necrosis, reactive oxygen species, varicose veins, venous closure, venous endothelial cells

## Abstract

**Introduction:**

Minimally invasive treatments for lower extremity varicose veins carry risks of thermal diffusion injury, nerve damage, ectopic embolism, and limited efficacy, highlighting the need for safer venous closure techniques. Microsecond irreversible electroporation (IRE) is a non-thermal ablation method whose mechanism of action on venous endothelium remains unclear.

**Methods:**

Using human umbilical vein endothelial cells (HUVECs) and a porcine hepatic portal vein model, we evaluated the killing efficacy, mechanism of action, and in vivo pathological changes induced by microsecond IRE at varying parameters.

**Results:**

IRE killed venous endothelial cells in a parameter-dependent manner: apoptosis predominated at 400 V/cm, while necrosis predominated at 800 V/cm with high pulse counts. IRE activated the intrinsic apoptotic pathway via reduced mitochondrial membrane potential, elevated ROS, and calcium overload, while promoting release of DAMPs (ATP, HMGB1, vWF). In the porcine portal vein model, IRE induced early inflammatory and microthrombotic changes while preserving overall vascular wall architecture.

**Discussion:**

Microsecond IRE achieves controllable, non-thermal venous endothelial injury and initiates signals necessary for venous closure, providing preclinical evidence for its development as a novel endovascular treatment for varicose veins.

## 1 Introduction

Varicose veins of the lower extremities are common benign diseases in vascular surgery. They are mainly caused by insufficiency of venous valves and blood reflux, leading to tortuosity and dilation of superficial veins. In the long term, they can cause skin lesions and ulcers, significantly affecting the quality of life of patients (Attaran et al., 2025). Currently, catheter-related thermal ablation (radiofrequency, laser) has become the standard minimally invasive surgical procedure for main venous reflux. It causes necrosis, fibrosis and closure of the vascular endothelium through thermal energy. However, thermal diffusion can easily cause burns to the skin and nerves, and may also increase the risk of postoperative pain, abnormal sensations (Jiang et al., 2024) and vascular perforation (Kanbur Üreyen et al., 2025). Chemical closure methods such as foam sclerosing agents have limitations such as ectopic embolism (Davies et al., 2022), allergic reactions (Wong et al., 2023), and poor efficacy for large veins (Bontinis et al., 2024). Therefore, there is an urgent need in clinical practice for a new minimally invasive technique that can precisely close the vascular endothelium and protect the surrounding normal tissues to the greatest extent (Lim et al., 2025).

Irreversible electroporation (IRE) is a non-thermal physical ablation technique that disrupts cell homeostasis through high-voltage microsecond pulsed electric fields, inducing apoptosis or necrosis of target cells (Jacobs et al., 2025). It is currently mainly used for ablation of malignant liver tumors and has been approved for clinical application. It has the advantages of precise targeting and minimal damage to surrounding normal tissues (Narayanan et al., 2024).

Based on the technical characteristics of IRE and the deficiencies of existing varicose vein treatments, it is of great value to explore its feasibility for endovascular treatment of varicose veins in the lower extremities. This study observed the damage effect of high-voltage microsecond pulses on vascular endothelial cells *in vitro* and the pathological changes of the portal vein in pigs, providing an experimental basis for subsequent endovascular applications and scheme optimization. It is expected to offer a safer and more physiologically consistent new strategy for vein closure. Although IRE has been maturely applied in the field of oncology, there is still a lack of systematic research on its biological effects on venous endothelium, the molecular mechanism of death, and the effect of venous closure *in vivo*. Clarifying the relevant injury mechanisms is the key theoretical basis for achieving the clinical transformation of this technology. This study aims to explore the killing efficacy of IRE on venous endothelial cells and the regulatory rules of apoptosis/necrosis through *in vitro* cell and *in vivo* animal experiments. It also intends to verify the pathological changes of the vascular wall in a porcine portal vein model, providing key preclinical evidence for IRE to become a new venous closure technology and offering directions for subsequent parameter optimization and device development.

## 2 Materials and methods

### 2.1 Microsecond IRE treatment parameters and cell processing

IRE treatment employs a steep pulsed electric field therapy system (Zhejiang Curaway Medical Technology Co., LTD.) to generate high-voltage microsecond-level pulses. *In vitro* cell experiments and *in vivo* portal vein experiments adjust the electric field intensity, pulse width, and pulse count according to the grouping, while maintaining the same basic pulse parameters.

Human umbilical vein endothelial cells (HUVEC) were purchased from Shanghai Fuheng Biotechnology CO., LTD. Special endothelial cell culture medium was used and cultured in Thermo Fisher Scientific HERACELL VIOS 160i CO_2_ incubator (37 ° C, 5% CO_2_). Cells from passages 3 to 8 were available for culture; however, to minimize passage-related variability in proliferative behavior and stress response, all main comparative experiments (cell viability assays, flow cytometry, Western blot, qRT-PCR, and DAMP quantification) were performed using cells within passages 4–6. Passage number was recorded for each experiment. The cells were digested with pancreatic enzymes without EDTA, centrifuged and resuspended. The density was adjusted to 1 × 10^6^ cells/mL. 800μL of the cell suspension was added to sterile shock cups (Bolo) spaced 4 mm apart and treated with different parameters of IRE. The untreated cells were used as blank controls (Con). Group setting: Blank control group; 400V/cm group 800V/cm group. For some cell activity experiments, 200V/cm and 600V/cm groups, as well as 20 μs, 50 μs, and 100 μs pulse width groups and 20, 40, 60, and 80 pulse groups were added. The parameters of each group were the same except for the target variable. The cells treated with IRE were further cultured for 12 h for subsequent detection.

### 2.2 Detection of cell viability, apoptosis and necrosis

Cell viability was determined by the CCK-8 method: After IRE treatment, HUVEC was seeded in 96-well plates at a rate of 1 × 10^5^ cells per well. After 12 h of incubation, 10μ l of CCK-8 solution was added and incubated for another 2 h. The absorbance at 450 nm was measured using a Swiss Dikon Spark® multi-functional microplate detector. Relative cell viability was calculated as: Viability (%) = (OD ∼ treatment∼ − OD ∼ background∼)/(OD ∼ control∼ − OD ∼ background∼) × 100%, where OD ∼ background ∼ represents the absorbance of medium-only wells without cells, and OD ∼ control ∼ represents the mean absorbance of untreated control wells.

Using PerCP/Cyanine5.5 AnnexinV/PI apoptosis detection kit necrosis and apoptosis: After 12 h of IRE treatment, cells were collected, washed twice with PBS, resuspended in binding buffer, and incubated at room temperature in the dark with 5 μL of LAnnexinV and PI. After resuspended in 400 μL of binding buffer, the cells were detected using a Beckman Coulter CytoFLEX series flow cytometer. Calculate the proportion of living cells, apoptotic cells and necrotic cells.

### 2.3 Detection of reactive oxygen species and mitochondrial membrane potential

Mitochondrial membrane potential was detected using the JC-1 Mitochondrial membrane potential Detection Kit (APExBIO): Collect the cells treated with IRE, resuspend them in JC-1 working solution, incubate at 37 ° C in the dark for 20 min, centrifuge at 4 ° C and discard the supernatant, and wash twice with pre-cooled staining buffer. The Beckman Coulter CytoFLEX series flow cytometer uses the FITC channel to detect the fluorescence intensity of JC-1 monomers and the PE channel to detect the fluorescence intensity of JC-1 polymers, reflecting changes in membrane potential.

The ROS level was detected using the DCFH-DA probe reactive oxygen species (ROS) detection kit (APExBIO): After IRE treatment, the cells were discarded from the culture medium, washed twice with PBS, and then 10μ m DCFH-DA was added. They were incubated at 37 ° C in the dark for 30 min. After washing, the relative fluorescence units were detected using a Swiss Diken Spark® multi-functional microplate detector at an excitation wavelength of 485 nm and an emission wavelength of 530 nm.

### 2.4 Detection of apoptosis-related protein and gene expression

Detection of apoptosis-related proteins using Westernblot: Cells were collected after IRE treatment, total protein was extracted, and the concentration was determined by BCA method. The cells were subjected to 4%–20% FuturePAGETM pre-prepared gel electrophoresis and transfer in a wet transfer tank. They were sealed at room temperature with 5% skimmed milk for 1 h. The sealing and antibody incubation process was gently shaken using a laboratory horizontal shaker or a MIULAB HS-25 shaker. Add BAX, Bcl-2, cleaved-caspase-3, cleaved-caspase-9, and β -actin primary antibodies, incubate overnight at 4 ° C. After TBST washing, add the corresponding HRP-labeled secondary antibody, incubate at room temperature for 1 h, and ECL luminescence development. The relative expression levels of proteins were detected using the Bio-RAD ChemiDoc MP all-round imaging system and quantified using ImageJ software.

Total RNA was extracted using the TRIzol method, lysis was assisted by Eppende ThermoMixer C constant temperature homogenizer, and cDNA was synthesized by reverse transcription using Applied Biosystems Veriti 96-well gradient PCR. Qrt-pcr was performed using PowerSYbrGreenRT-PCR reagent (Takara, RR820A) in the Applied Biosystems StepOnePlus™ real-time fluorescence quantitative PCR instrument, with GAPDH as the internal reference. The expressions of BAX and Bcl-2 mrna were detected, and the relative expression levels were calculated by the 2^−ΔΔCt^ method.

### 2.5 Detection of intracellular Ca^2+^, ATP, HMGB1, vWF and extracellular pH

Intracellular Ca^2+^ was detected using the FluO-4 calcium ion fluorescence assay kit (Wuhan Elairite Biotechnology Co., LTD.), with excitation/emission wavelengths of 490nm/525 nm. The relative fluorescence intensity was determined using the Swiss Diken Spark® multi-functional microplate detector.

The ATP concentration of the cell supernatant was detected by the ATP content chemiluminescence test kit (Wuhan Elairite Biotechnology Co., LTD.), and the fluorescence intensity was measured and the concentration was calculated by the Swiss Diken Spark® multi-functional microplate detector.

The concentrations of HMGB1 and vWF in the cell supernatant were detected by ELISA kits. The absorbance at 450 nm was measured by the Swiss Diken Spark® multi-functional microplate detector. The contents were calculated in combination with the standard curve.

Extracellular fluid was collected before IRE treatment and at 0.5h, 6h and 12 h after treatment respectively, and the pH value changes were detected by a pH meter.

### 2.6 Portal vein IRE treatment in experimental animals and *in vivo*

All animal experiments were approved by the institution’s animal ethics committee (Ethics Number: 20250827-13). The experimental pigs were raised in standardized animal rooms, freely fed and drank water, and adapted to the environment for 3 days before the experiments were conducted.

The steep pulse treatment equipment consistent with the cell experiment was adopted, in combination with the one-time pulse dilation ablation catheter NKKA1050N (outer diameter 8Fr, total length 180 cm, working length 50 mm, maximum dilation diameter 10 mm). Under ultrasound guidance, coaxial puncture of the portal vein was performed, and an ablation catheter was implanted. The IRE parameters were applied: 800V, 100 μs, and 80 pulses. Three days after the operation, portal vein tissue was collected for pathological examination. Three pigs (n = 3) were included in the study; each animal underwent a single IRE treatment session, and one portal vein specimen was collected per animal. No animals or specimens were excluded from the analysis. The study did not include a sham-operated control group (catheter insertion without IRE pulse delivery); the potential contribution of mechanical catheter trauma to the observed histological changes therefore cannot be fully excluded, and this is acknowledged as a limitation.

### 2.7 Tissue and cell collection and processing

*In vitro* cells: After IRE treatment, cells were collected by trypsin digestion for protein and RNA extraction as well as flow cytometry detection. Some cells were fixed with electron microscope fixative after 12 h of culture and then used for transmission electron microscope observation.

*In vivo* tissues: Portal vein tissues were dissected immediately after IRE treatment, fixed with 4% paraformaldehyde, embedded in paraffin, and continuously sectioned at 5 μm for H&E staining. Samples that were not processed in time should be rapidly frozen and stored.

### 2.8 Portal vein pathological examination (H&E staining)

The portal vein tissue sections were dewaxed, stained with hematoxylin-eosin, dehydrated, and sealed with transparent and neutral gum. The vascular wall structure, endothelial cell morphology, lumen and surrounding tissue changes were observed under an optical microscope, and morphological quantitative analysis was performed by an image analysis system.

### 2.9 Transmission electron microscopy observation

After being treated with IRE and cultured for 12 h, HUVEC cells were fixed with electron microscopy fixative, subjected to gradient dehydration, embedding, ultrathin sectioning and staining. The cell morphology, cell membrane, and structural changes of organelles such as mitochondria and nucleus were observed by transmission electron microscopy.

### 2.10 Statistical analysis

Data analysis was conducted using SPSS 26.0 statistical software. Measurement data are expressed as mean ± standard deviation (SD). Prior to group comparisons, normality of each dataset was assessed using the Shapiro-Wilk test. For datasets meeting normality assumptions, one-way ANOVA was applied for multi-group comparisons, followed by Tukey’s honest significant difference (HSD) *post hoc* test for pairwise comparisons. All error bars in figures represent SD of at least three independent experiments. Statistical significance thresholds were defined as: *P < 0.05, **P < 0.01, ***P < 0.001.

## 3 Results

### 3.1 Effects of different IRE electric field parameters on the viability, apoptosis and necrosis of vascular endothelial cells

**FIGURE 1:**
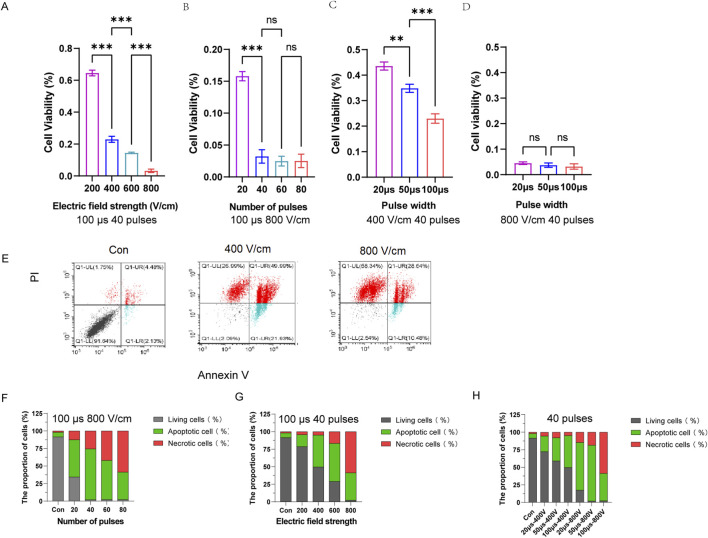
Effects of different microsecond IRE parameters on cell viability, apoptosis, and necrosis of vascular endothelial cells. Note: Con is the blank control group, 400V is the 400V /cm group, and 800V is the 800V /cm group. **(A–D)** Effects of IRE treatment with different electric field intensities, pulse numbers and pulse widths on the viability of HUVEC cells (ns, no statistically significant difference (P > 0.05); *P ≤ 0.05, statistically significant difference; **P ≤ 0.01, highly significant difference; ***P ≤ 0.001, extremely significant difference). **(E)** Annexin V/PI double-staining flow cytometry was used to detect the apoptosis and necrosis of HUVEC after treatment with different parameters of IRE. **(F,G,H)** Statistics of the proportions of living cells, apoptotic cells and necrotic cells based on flow cytometry results.

At fixed 100 μs pulse width, 40 pulses, and electric field strengths of 200–800 V/cm, HUVEC cell viability showed a highly significant dose-dependent decrease (P < 0.001) (Figure 1A), with viability of 64.5% ± 3.2% in the 200 V/cm group, 21.8% ± 2.1% in the 400 V/cm group, 13.6% ± 1.5% in the 600 V/cm group, and 1.8% ± 0.7% in the 800 V/cm group 1.8% ± 0.7%, and the difference between 200 V/cm and the remaining groups was highly significant (P < 0.001). Fixing 800 V/cm and 100 μs pulse width, the 20-pulse group was 15.3% ± 1.8% more vigorous than the 40–80-pulse group (2.5% ± 0.6%, 1.9% ± 0.5%, 1.7% ± 0.4%, P0.05) (Figure 1B). Cell viability decreased significantly (P < 0.01 or P < 0.001) at fixed 400 V/cm, 40 pulses, and pulse widths of 20–100 μs (Figure 1C), with 44.2% ± 2.7% in the 20 μs group, 34.8% ± 2.3% in the 50 μs group, and 21.5% ± 1.9% in the 100 μs group; and at fixed 800 V/cm, 40 pulses, the change in pulse width had no effect on the viability had no effect (P > 0.05), and the viability of all groups was lower than 2.1% ± 0.8% (Figure 1D). Annexin V/PI double staining showed (Figures 1E,G,I) that under the fixed conditions, the increase in the number of pulses and the increase in the field strength caused a decrease in the proportion of live cells, a rise in the proportion of apoptosis and then a decrease in the proportion of necrosis; the 80 pulses, 800 V/cm group had about 2.1%–2.3% of live cells, and the necrosis accounted for about 58.6%–59.2% (Figures 1F,H). The prolongation of pulse width at 400 V/cm promoted apoptosis with a necrosis percentage of <5%; at 800 V/cm the live cells in each pulse width group were <3% with a necrosis percentage of 55.2%–61.3% (Figure 1J).

### 3.2 Effects of microsecond IRE on HUVEC cell morphology, ultrastructure, mitochondrial membrane potential, ROS level and intracellular pH value

**FIGURE 2:**
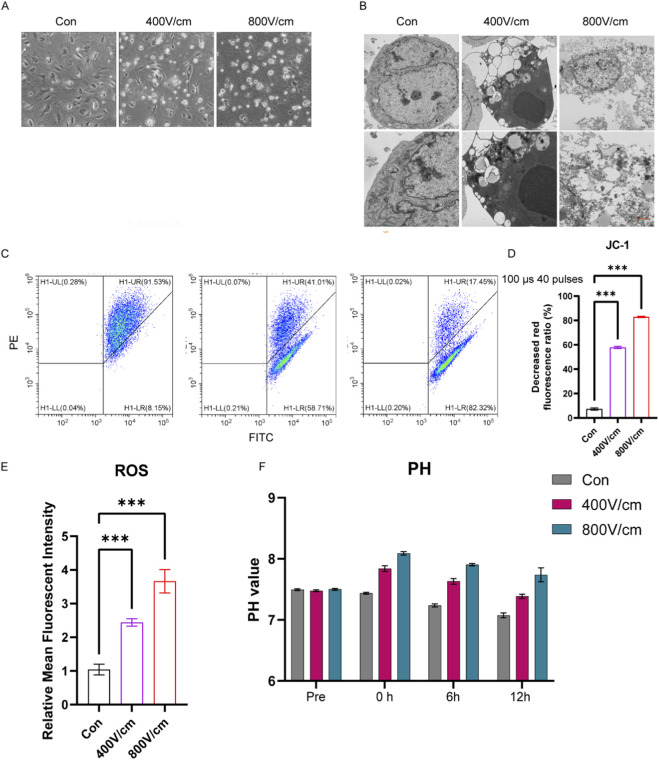
Effects of microsecond IRE on HUVEC cell morphology, ultrastructure, mitochondrial membrane potential, ROS level, and extracellular pH. Note: **(A)** Light microscopy cell morphology; **(B)** Transmission electron microscopy cell ultrastructure; **(C,D)** JC-1 staining to detect mitochondrial membrane potential (**(C)** graph is a representative scatter plot, **(D)** graph is a quantitative statistic of the proportion of cells with decreased membrane potential); **(E)** Intracellular ROS levels; **(F)** Extracellular pH changes at indicated time points.

Under the light microscope, HUVEC in the control group grew in the shape of a shuttle and attached to the wall, the cells in the 400 V/cm group were crumpled and the gap was enlarged, and a large number of cells in the 800 V/cm group were rounded and detached (Figure 2A). Transmission electron microscopy showed that the ultrastructure of the cells in the control group was normal, and the 400 V/cm group showed typical apoptotic features, such as chromatin margination, cytoplasmic vacuolization, and intact cell membranes; while the 800 V/cm group showed obvious necrotic features, such as rupture of cell membranes, cytoplasmic egress, and dispersion of chromatin (Figure 2B). The results of JC-1 staining showed that high mitochondrial membrane potential dominated in the control group, and the proportion of cells with low membrane potential increased in the 400 V/cm and 800 V/cm groups, and the quantitative statistics showed that the proportion of membrane potential decreased in both groups was significantly higher than that in the control group (P < 0.001) (Figures 2C,D). The ROS assay showed that compared with the control group, the relative fluorescence intensity of ROS in the 400 V/cm and 800 V/cm groups was significantly higher (P < 0.001), and the ROS level was higher in the 800 V/cm group (Figure 2E). The extracellular pH values showed that there was no significant difference in the pH values of the groups before treatment; the pH values of the control group gradually decreased at 0 h, 6 h and 12 h after treatment, while the pH values of the 400 V/cm and 800 V/cm groups were higher than those of the control group, and the increase was more obvious in the 800 V/cm group (Figure 2F).

### 3.3 Effect of microsecond IRE on the expression of apoptosis-related genes and proteins in HUVEC cells

**FIGURE 3:**
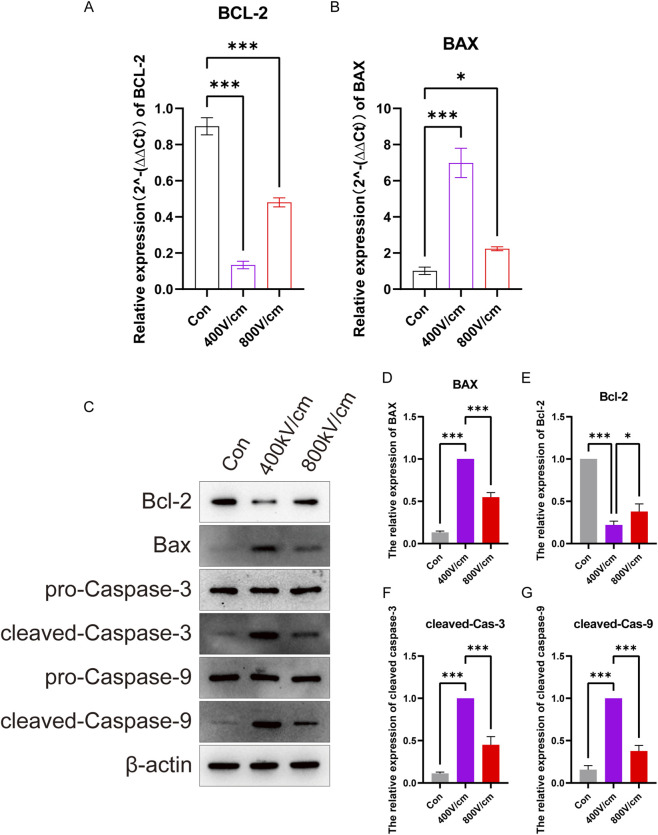
Effects of microsecond IRE on the expression of apoptosis-related genes and proteins in HUVEC cells. Note: **(A,B)** qRT-PCR to detect the relative mRNA expression levels of Bcl-2 and BAX **(C)** Western blot to detect the expression bands of apoptosis-related proteins (Bcl-2, Bax, pro-Caspase-3, cleaved-Caspase-3, pro-Caspase-9, cleaved- Caspase-9) expression bands, and β-actin was the internal reference **(D–G)** Quantitative gray scale analysis results of Western blot strips for relative expression levels of BAX, Bcl-2, cleaved-Caspase-3, cleaved-Caspase-9 proteins, respectively. Con is the control group, 400 V/cm and 800 V/cm are the different field strength microsecond steep pulse treatment groups; *P < 0.05, ***P < 0.001, compared with the corresponding groups.

The qRT-PCR results showed that the relative expression levels of Bcl-2 mRNA were significantly lower in the 400 V/cm and 800 V/cm groups compared with the control group (Con) (P < 0.001), and the expression level in the 400 V/cm group was lower than that in the 800 V/cm group (Figure 3A); Compared with the control group, the relative expression levels of BAX mRNA were significantly higher in the 400 V/cm and 800 V/cm groups (P < 0.001), and the expression level in the 400 V/cm group was higher than that in the 800 V/cm group (Figure 3B). Western blot results showed that the signal intensity of Bcl-2 protein bands was the strongest in the control group, the weakest in the 400 V/cm group, and the middle in the 800 V/cm group; the signal intensity of BAX, cleaved-Caspase-3, cleaved Caspase-9 protein bands were weakest in the control group, strongest in the 400 V/cm group, and intermediate in the 800 V/cm group; pro-Caspase-3, pro-Caspase-9, and endoglin were weakest in the control group, strongest in the 400 V/cm group, and intermediate in the 800 V/cm group. Caspase-3, cleaved-Caspase-9 and β-actin were the weakest in the control group, the strongest in the 400 V/cm group and the middle in the 800 V/cm group (Figure 3C). Protein quantification statistics showed that the relative expression levels of BAX proteins were significantly higher in the 400 V/cm and 800 V/cm groups compared with the control group (P < 0.001), and the 400 V/cm group was higher than the 800 V/cm group (Figure 3D); Compared with the control group, the relative expression levels of Bcl-2 protein were significantly lower in the 400 V/cm and 800 V/cm groups (P < 0.001), with the 400 V/cm group being lower than that of the 800 V/cm group (Figure 3E); Compared with the control group, the relative expression levels of cleaved-Caspase-3 proteins were significantly higher in the 400 V/cm and 800 V/cm groups (P < 0.001), and the 400 V/cm group was higher than that of the 800 V/cm group (Figure 3F); Compared with the control group, the relative expression levels of cleaved-Caspase-9 proteins were significantly higher in the 400 V/cm and 800 V/cm groups (P < 0.001), and higher in the 400 V/cm group than in the 800 V/cm group (Figure 3G).

### 3.4 Microsecond IRE induces calcium overload and damage-associated molecular patterns (DAMPs) release in HUVEC cells

**FIGURE 4:**
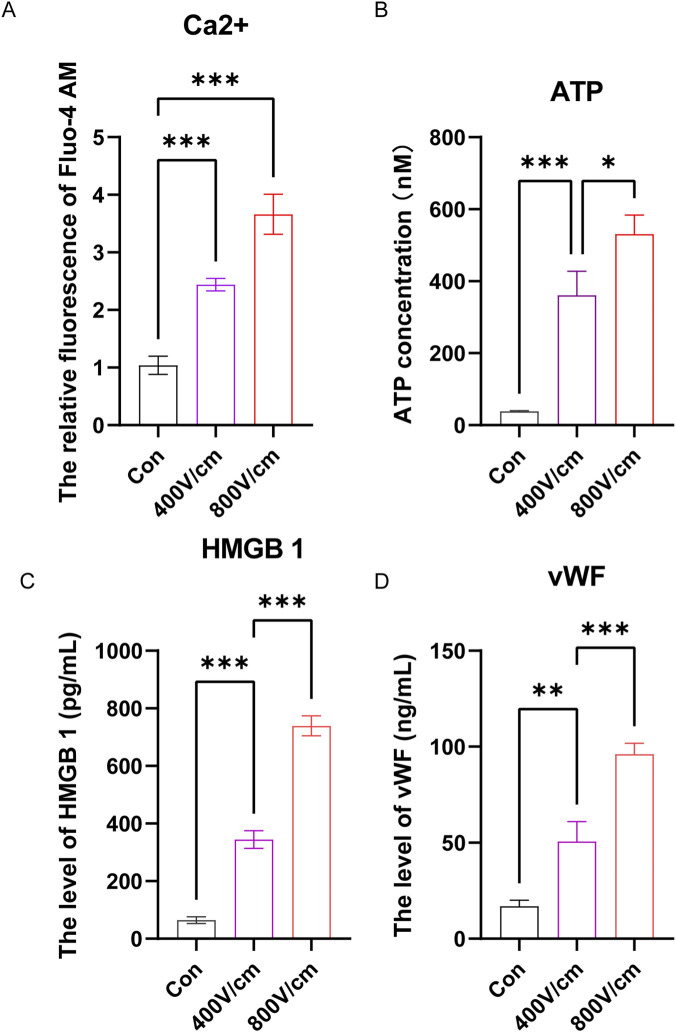
Microsecond IRE induced calcium overload and damage-associated molecular patterns (DAMPs) release in HUVEC cells. Note **(A)** Fluo-4 AM staining to detect the relative fluorescence intensity of intracellular Ca^2+^ **(B)** Detection of extracellular ATP concentration **(C)** ELISA to detect the level of HMGB1 in the supernatant of the cells **(D)** ELISA to detect the level of vWF in the supernatant of the cells. Con is the control group, and 400 V/cm, 800 V/cm are the groups treated with different field strengths. Con is the control group, 400 V/cm and 800 V/cm are the different field strengths of the steep pulse treatment group; *P < 0.05, **P < 0.01, ***P < 0.001, compared with the corresponding groups.

The results of intracellular Ca^2+^ assay showed that the relative fluorescence intensity of intracellular Ca^2+^ was significantly higher in the 400 V/cm and 800 V/cm groups compared with the control group (Con) (P < 0.001), and it was higher in the 800 V/cm group than in the 400 V/cm group (Figure 4A). The results of extracellular ATP concentration assay showed that compared with the control group, the extracellular ATP concentration in the 400 V/cm and 800 V/cm groups were significantly higher (P < 0.001 for the 400 V/cm group and P < 0.05 for the 800 V/cm group), and the 800 V/cm group was higher than the 400 V/cm group (Figure 4B). The results of cell supernatant HMGB1 levels showed that compared with the control group, the levels of HMGB1 in cell supernatants of the 400 V/cm and 800 V/cm groups were significantly higher (P < 0.001), and the 800 V/cm group was higher than that of the 400 V/cm group (Figure 4C). The results of vWF levels in cell supernatants showed that compared with the control group, the levels of vWF in the supernatants of cells in the 400 V/cm and 800 V/cm groups were significantly elevated (P < 0.01 in the 400 V/cm group and P < 0.001 in the 800 V/cm group) and were higher in the 800 V/cm group than those in the 400 V/cm group (Figure 4D).

### 3.5 Pathologic effects of microsecond IRE on the portal vein in pigs (H&E staining)

**FIGURE 5:**
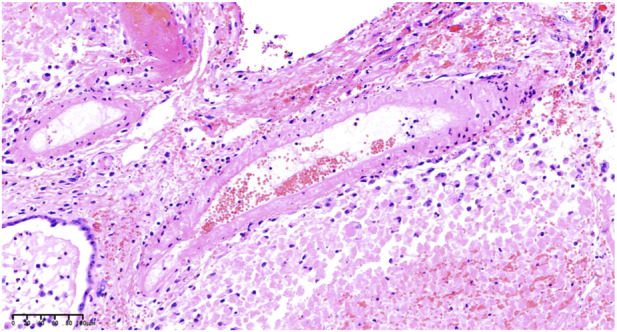
Pathomorphologic observation of HE staining of porcine portal vein tissue after microsecond IRE treatment.

Microscopic HE staining showed that the lumen of porcine portal vein was still intact, but the continuity of endothelial cells was interrupted and detached in some areas, and the subendothelial layer was exposed; the smooth muscle layer of the vascular wall showed varying degrees of vacuolar degeneration, cellular crumpling, deep staining of cellular nuclear condensation, and some smooth muscle cells were not clearly defined; erythrocytes were seen to accumulate and microthrombus-like changes could be seen in the lumen; the mesenchymal stroma around the blood vessels was spongy and oedematous, accompanied by scattered lymphocytes and monocytes. Overall, it showed microsecond steep pulse-induced vascular endothelial and smooth muscle cell injury, with cellular crumpling and nuclear consolidation as the main manifestations, accompanied by interstitial edema and early inflammatory reaction in some areas, without extensive necrosis, rupture of the vascular wall or massive hemorrhagic changes (Figure 5).

## 4 Discussion

Clinical treatment of varicose veins of the lower extremities has long faced problems such as thermal diffusion damage (Jiang et al., 2024)and limited efficacy of chemical closure (Bontinis et al., 2024), and exploring new closure techniques that are precise, minimally invasive and cause little damage to surrounding tissues is a key need to improve the prognosis of patients and enhance the safety of treatment (Lim et al., 2025). IRE as a non-thermal physical ablation technique, has been proved to be safe and effective for ablation of malignant solid tumors of the liver (Narayanan et al., 2024). Its advantage of destroying the homeostasis of target cells without damaging the surrounding normal tissues provides important theoretical support for its expansion to endovenous treatment of varicose veins of the lower extremities (Jacobs et al., 2025). In the present study, the killing efficacy of microsecond IRE on venous endothelial cells, the death mode and the pathological changes of the vascular wall were systematically investigated through the *in vitro* HUVEC model and the *in vivo* porcine portal vein experiments, which advances the mechanistic characterization of microsecond IRE in venous tissues and provides preliminary preclinical evidence supporting its further investigation as a potential endovascular treatment strategy. It should be noted, however, that HUVECs derived from normal umbilical vein do not recapitulate the pathological endothelial phenotype present in varicose vein tissue—including chronic exposure to elevated hydrostatic pressure, disturbed shear stress, and oxidative remodeling—and the translational relevance of these findings to clinical varicose vein treatment should therefore be interpreted with caution pending validation in disease-relevant endothelial cell models.

In this study, the *in vitro* experiments clarified the regulation pattern of IRE on HUVEC cell activity and death mode by setting IRE treatments with different electric field strengths, pulse widths, and number of pulses, which is both consistent with the findings of IRE in the field of oncology and the specificity for venous endothelial cells. In tumor research, IRE often achieves tissue inactivation by inducing apoptosis or necrosis of target cells, whereas in this study, we found that the killing effect of IRE on HUVEC cells had an obvious parameter dependence: with the increase of the electric field intensity, the prolongation of the pulse width, and the number of pulses, the relative activity of the cells decreased significantly, and the proportion of the necrotic cells were significantly increased (combined with the results of the cellular activity assay and the flow cytometry results), which suggests that This suggests that IRE can achieve controllable damage to venous endothelial cells by precisely regulating the pulse parameters, which provides an experimental basis for optimizing the parameters according to the diameter of vein and the degree of lesion in clinical treatment. The expression of apoptosis-related proteins BAX, cleaved-Caspase-3 and cleaved-Caspase-9 was significantly upregulated, the expression of anti-apoptosis protein Bcl-2 was downregulated, and the relative expression of BAX gene was synchronously increased in HUVEC cells after IRE treatment, confirming that apoptosis is one of the most important pathways in IRE-induced endothelial cells. Pathway is an important mechanism of IRE-induced death of venous endothelial cells, which is consistent with the molecular mechanism of IRE-induced apoptosis of tumor cells, but is fundamentally different from the mode of action of thermal ablation technology that induces endothelial necrosis through heat, and also explains why IRE can maximally preserve the normal tissues around the blood vessels while accurately damaging the endothelium.

An apparent paradox in the protein expression data warrants explicit discussion: BAX, cleaved caspase-3, and cleaved caspase-9 showed higher band intensities in the 400 V/cm group than in the 800 V/cm group, despite the 800 V/cm condition producing greater overall cell death. We attribute this pattern to the high proportion of necrotic death (∼58–59%) at 800 V/cm. Necrosis involves plasma membrane rupture, leading to leakage of cytoplasmic contents—including β-actin used for normalization—into the culture medium. Total protein recovered from cell lysates in the 800 V/cm group is therefore substantially reduced, causing systematic underestimation of all protein band intensities, including pro-apoptotic markers, even after β-actin normalization. This normalization artifact does not contradict the conclusion that apoptosis predominates at 400 V/cm; rather, it is an expected consequence of extensive membrane disruption at 800 V/cm. The same logic applies to the mRNA data: the relatively higher BCL2 mRNA in the 800 V/cm group likely reflects incomplete transcriptional reprogramming, as rapid membrane disruption at high field strength may preempt the full BCL2 downregulation that characterizes the mature intrinsic apoptotic program at 400 V/cm.

Mitochondrial dysfunction and oxidative stress are important regulatory aspects of apoptosis. In this study, we further revealed the potential molecular mechanism of IRE-induced apoptosis in HUVEC cells by detecting ROS content, mitochondrial membrane potential and intracellular Ca^2+^ concentration. The results showed that IRE-treated HUVEC cells had significantly elevated intracellular ROS content, markedly decreased mitochondrial membrane potential, and abnormally elevated intracellular Ca^2+^ concentration, which is consistent with previous findings of IRE-induced damage in tumor cells-that ROS overproduction can trigger an oxidative stress reaction, destroying mitochondrial membrane integrity and leading to mitochondrial dysfunction, whereas a decrease in mitochondrial membrane potential can further initiate endogenous apoptotic pathways, while an imbalance in intracellular Ca^2+^ homeostasis can activate calcium-dependent apoptosis-related enzymes, accelerating the process of apoptosis (Rajagopalan et al., 2024; Polajžer et al., 2024; Kulbacka et al., 2023). Elevated extracellular vWF may arise from two mechanistically distinct sources with different implications for the proposed venous closure mechanism. First, active secretion from Weibel-Palade bodies in sublethal or stressed-but-viable endothelial cells, driven by the intracellular Ca^2+^ overload documented in this study, would engage physiological platelet adhesion and organized thrombosis cascades—a process more directly relevant to functional venous closure. Second, passive leakage from cells undergoing necrotic membrane rupture releases vWF without the spatial organization required for targeted platelet recruitment. Given that the 400 V/cm condition is associated with predominantly apoptotic death and a larger fraction of residual viable cells, active Weibel-Palade body secretion is likely the dominant vWF source at lower field strengths, whereas passive leakage predominates at 800 V/cm where necrosis accounts for ∼58–59% of cell death. These mechanistic distinctions are speculative in the absence of experiments directly distinguishing the two pathways, and future studies should address this using Weibel-Palade body co-staining or vWF multimer profiling. Based on the DAMP release profile observed—elevated extracellular ATP, HMGB1, and vWF—it is reasonable to propose as a hypothesis that microsecond IRE may engage inflammasome-mediated inflammatory signaling, drawing a potential parallel to mechanisms described for H-FIRE (Da et al., 2024). H-FIRE has been reported to activate the NLRP3 inflammasome, promote gasdermin D cleavage, and drive pyroptotic maturation of IL-1β and IL-18. However, whether microsecond IRE activates the same pathway under the parameters used in the present study remains entirely untested. We did not measure LDH release, gasdermin D cleavage, or mature IL-1β/IL-18 secretion in this study, and the mechanistic parallel with H-FIRE should not be taken as established. This hypothesis requires future investigation using NLRP3 inhibition (e.g., MCC950), gasdermin D immunoblotting, and cytokine multiplex assays to determine whether microsecond IRE triggers pyroptotic signaling in venous endothelial cells.

*In vivo* experiments by IRE treatment of porcine portal vein visualized 3-day post-pulse pathological changes in the vessel wall, providing direct evidence for the feasibility of IRE-induced venous closure. As an important part of the venous system, the portal vein has a certain similarity in the structure of its vessel wall with the superficial veins of the lower limbs, both of which are composed of endothelial cells, smooth muscle cells and connective tissues, so the results of the present study can initially reflect the characteristics of the action of IRE on venous tissues. The present study demonstrates that the applied IRE parameters (800 V, 100 μs, 80 pulses) induced histological changes in the porcine portal vein consistent with injury to the endothelial layer and smooth muscle cells—evidenced by endothelial discontinuity, vacuolar degeneration, and nuclear condensation on H&E—alongside early inflammatory infiltration and microthrombotic changes, without transmural necrosis or vascular rupture. It should be noted that cell-type identification in the present study relied on H&E morphology and anatomical location alone; immunohistochemical confirmation using CD31 or VE-cadherin (endothelium), α-SMA (smooth muscle), CD68 (macrophages), and fibrin or platelet markers (thrombosis) would be required to definitively confirm these assignments and should be prioritized in future studies. The preserved vascular wall architecture at 3 days post-IRE is an encouraging early finding, but 3 days represents an acute time point only. Delayed vascular remodeling, progressive wall changes, or incomplete closure at later time points cannot be excluded on the basis of these data alone, and longitudinal histological assessment is required. In addition, as noted in the Methods, no sham catheter control was included; the contribution of mechanical insertion trauma to the observed histological changes therefore cannot be fully excluded. Lower extremity veins and portal veins are both vascular tissues composed of endothelial cells and smooth muscle cells, and the targeted injury mechanism of IRE on portal veins can be initially migrated to the closure of lower extremity veins. It should be acknowledged that while IRE is classified as a non-thermal ablation technique—with cell death attributed primarily to electrical membrane disruption rather than heat-induced protein denaturation—this designation does not imply zero temperature elevation. Joule heating during delivery of high-voltage microsecond pulses is a real physical phenomenon. In the present study, tissue temperature was not directly measured during IRE delivery, and transient temperature rise under the applied parameters (800 V, 100 μs, 80 pulses) cannot be excluded. Future studies should incorporate direct thermometry during pulse delivery to confirm that temperature elevations remain below the threshold for thermal tissue injury. IRE does not require high temperature, which avoids the damage of the surrounding tissues caused by traditional thermal ablation, and it is adapted to the anatomical characteristics of the fragile tissues such as the nerves and the skin around the lower extremity veins, which reduces the risk of complication.

Although this study provides initial mechanistic evidence for IRE-induced venous endothelial injury, several important limitations must be acknowledged. First, the *in vitro* experiments employed commercially purchased normal HUVECs, which do not recapitulate the pathological endothelial phenotype of varicose vein tissue—including chronic exposure to elevated hydrostatic pressure, disturbed shear stress, and oxidative remodeling—that meaningfully alters BCL2/BAX balance, proliferative behavior, and cellular stress thresholds. The parameter dependencies identified here may therefore differ in endothelial cells derived from varicose vein specimens, and direct translational inferences should be made with caution. Future studies using primary endothelial cells isolated from varicose vein tissue are required to confirm the clinical relevance of these findings. Second, the assignment of cell death mode as apoptosis (400 V/cm) or necrosis (800 V/cm) was based on Annexin V/PI staining and TEM morphology, which are suggestive but not definitive: late apoptotic and secondary necrotic populations are both Annexin V+/PI+, and TEM morphology cannot distinguish primary necrosis from secondary necrotic transition. Confirmation requires caspase inhibitor rescue experiments (e.g., Z-VAD-FMK), TUNEL assay, or cytochrome c release. Additionally, RIPK3 and MLKL phosphorylation should be evaluated at 800 V/cm to exclude necroptosis as a contributing mechanism. Third, tissue temperature was not measured during IRE delivery; Joule heating during high-voltage microsecond pulse delivery is a recognized physical phenomenon, and the non-thermal classification of the observed cell death at the applied parameters cannot be confirmed without direct thermometry data. Future studies should incorporate temperature monitoring. Fourth, the *in vivo* component was limited to n = 3 animals at a single time point (3 days post-IRE), used qualitative H&E assessment only, and lacked a sham catheter control group; cell-type specificity was inferred from morphology and anatomical location rather than confirmed by immunohistochemistry. Multi-marker IHC (CD31/VE-cadherin, α-SMA, CD68, fibrin/platelet markers), semi-quantitative histological scoring, a sham catheter control, and longitudinal time points are all needed to substantiate the *in vivo* claims. Fifth, an animal model of lower extremity varicose veins was not established, the optimal parameter combination for clinical endovenous application was not defined, and electrode placement geometry for clinical treatment was not simulated, limiting direct clinical translation. Finally, the repair and remodeling mechanisms following IRE-induced venous endothelial injury, and potential synergies with existing techniques such as foam sclerotherapy, remain to be explored in future work.

Combining the existing research progress and the results of this study, IRE, as a nonthermal physical ablation technique, has a broad application prospect in the endovenous treatment of varicose veins of the lower extremities. Compared with previous applications of IRE in the field of oncology, this study focuses on venous endothelial cells for the first time, clarifies the killing mechanism and parameter dependence of IRE on venous endothelial cells, and fills the research gaps in the application of IRE in the field of venous diseases. Future research can focus on the following: first, establish an animal model of varicose veins in the lower limbs, simulate the clinical lesion characteristics, and assess the long-term effect of IRE-induced venous closure and its effect on valve function; second, optimize the IRE pulse parameters, and formulate a personalized therapeutic parameter scheme by combining the characteristics of the clinical venous lesions; and third, deeply investigate the repair mechanism of IRE-induced venous endothelial damage and the local inflammatory reaction’s Thirdly, to deeply investigate the repair mechanism of IRE-induced venous endothelial damage and the regulation law of local inflammatory response, in order to provide theoretical basis for the promotion of physiological closure of blood vessels; fourthly, to carry out the research on the combined treatment of IRE with foam sclerosant and checkpoint inhibitor, *etc.*, in order to further enhance the therapeutic effect and reduce the complications; fifthly, to promote the research on the preclinical safety and efficacy, so as to provide solid experimental support for the transformation of IRE into a new minimally invasive treatment for varicose veins of lower limbs.

In summary, the present study demonstrated that IRE can induce apoptosis of venous endothelial cells, trigger mitochondrial dysfunction and oxidative stress, achieve controlled damage to the venous endothelium, and effectively act on the venous wall *in vivo*, which provides an important preclinical experimental basis for the application of endovenous treatment of varicose veins of the lower extremities. With the advantages of precision, minimally invasive, and slight damage to the surrounding tissues, IRE is expected to overcome the limitations of the existing treatments and provide a new treatment strategy that is safer and more in line with the physiological closure process for patients with varicose veins of the lower extremities, which has an important clinical translational value and application prospect.

## Data Availability

The original contributions presented in the study are included in the article/supplementary material, further inquiries can be directed to the corresponding author.
